# Meta-analysis overstates benefit of antidepressant combination therapy with α2-antagonists and reuptake inhibitors in major depression

**DOI:** 10.3389/fpsyt.2022.1053530

**Published:** 2022-10-31

**Authors:** Kevin P. Kennedy

**Affiliations:** Department of Psychiatry and Biobehavioral Sciences, University of California, Los Angeles, Los Angeles, CA, United States

**Keywords:** antidepressants (AD), combination (combined) therapy, meta-analysis, α2-antagonist, major depression (MDD), selective serotonergic reuptake inhibitors (SSRI)

Earlier this year, Henssler et al. (1) published an important meta-analysis exploring the treatment of major depression with combination antidepressants compared to monotherapy. They concluded that combination therapy of a reuptake inhibitor (SSRI, SNRI, or tricyclic antidepressant) with an α2-antagonist is more effective than antidepressant monotherapy, with a SMD of 0.37. They found no benefit from combination therapy involving bupropion. Given the modest response and remission rates from antidepressant monotherapy, this finding has the potential to widely influence clinical practice.

There are reasons clinicians should be cautious about this result. The authors included 18 studies in their analysis of α2-antagonists, with 10 studies on mirtazapine, six on mianserin, and two on trazodone [which may have only modest α2-antagonism at the doses used in these studies (2, 3)]. The majority of these studies were quite small, but five were robust and included 1,647, 665, 480, 293, and 204 subjects. All five of these large studies were negative [or, in one case (4), potentially statistically significant in one arm but clearly clinically insignificant and negative as a whole: there was no difference between mirtazapine monotherapy and combination; a clinically-insignificant 0.99 PHQ-9 points difference between sertraline monotherapy and mirtazapine combination at 9 weeks; and no difference between any arm at 25 weeks]. By contrast, the five largest positive studies included only 105, 103, 61, 60, and 47 subjects, with 77, 32, 21, 30, and 21 subjects in the combination medication arms, respectively. Four of the 10 positive studies were rated as high or unknown risk of bias.

When numerous large, well-designed designed trials consistently find no benefit to α2-antagonist combination therapy, we should be cautious to accept positive results from a meta-analysis whose effect appears to be driven by far smaller studies, especially when the results are highly heterogeneous (*I*^2^ = 80.6%) and reflect significant small-study effects and publication bias (5). The authors' effort to correct for publication bias resulted in a very modest effect size of 0.19 with marginal statistical significance (95% CI, 0.01–0.36).

While there is a reasonable debate regarding whether meta-analyses or large high-quality RCTs should guide clinical practice when their findings differ, this is a case where there are multiple large studies across different settings with consistent negative findings—and not a single comparable study with positive findings. Most clinicians would intuitively recognize that small positive aggregate effects driven by highly heterogenous small studies should not override the clear outcomes of multiple large RCTs. They would likely interpret this as small positive findings failing to replicate when applied to larger samples.

This may be an instance where a common-sense reading of the literature should prevail, and where the over-valuation of small studies with large effect sizes in a random-effects meta-analysis may obfuscate rather than illuminate the true effects of a medication. When there are numerous large negative trials and not a single comparable positive study showing clinically-significant benefit from combination antidepressant therapy with α2-antagonists, clinicians should be hesitant to prescribe this treatment.

## Author contributions

The author confirms being the sole contributor of this work and has approved it for publication.

## Conflict of interest

The author declares that the research was conducted in the absence of any commercial or financial relationships that could be construed as a potential conflict of interest.

## Publisher's note

All claims expressed in this article are solely those of the authors and do not necessarily represent those of their affiliated organizations, or those of the publisher, the editors and the reviewers. Any product that may be evaluated in this article, or claim that may be made by its manufacturer, is not guaranteed or endorsed by the publisher.
